# Formulation and Evaluation of a Self-Microemulsifying Drug Delivery System of Raloxifene with Improved Solubility and Oral Bioavailability

**DOI:** 10.3390/pharmaceutics15082073

**Published:** 2023-08-02

**Authors:** Muhammad Mohsin Ansari, Dang-Khoa Vo, Ho-Ik Choi, Jeong-Su Ryu, Yumi Bae, Nadeem Irfan Bukhari, Alam Zeb, Jin-Ki Kim, Han-Joo Maeng

**Affiliations:** 1 Riphah Institute of Pharmaceutical Sciences, Riphah International University, Islamabad 44000, Pakistan; 2 College of Pharmacy, Gachon University, 191 Hambakmoe-ro, Yeonsu-gu, Incheon 21936, Republic of Korea; 3 College of Pharmacy, Institute of Pharmaceutical Sciences and Technology, Hanyang University, 55 Hanyangdaehak-ro, Sangnok-gu, Ansan 15588, Republic of Korea; 4 Punjab University College of Pharmacy, University of Punjab, Lahore 54590, Pakistan

**Keywords:** raloxifene hydrochloride, BCS class II, solubility, dissolution, oral bioavailability, SMEDDS

## Abstract

Poor aqueous solubility and dissolution limit the oral bioavailability of Biopharmaceutics Classification System (BCS) class II drugs. In this study, we aimed to improve the aqueous solubility and oral bioavailability of raloxifene hydrochloride (RLX), a BCS class II drug, using a self-microemulsifying drug delivery system (SMEDDS). Based on the solubilities of RLX, Capryol 90, Tween 80/Labrasol ALF, and polyethylene glycol 400 (PEG-400) were selected as the oil, surfactant mixture, and cosurfactant, respectively. Pseudo-ternary phase diagrams were constructed to determine the optimal composition (Capryol 90/Tween 80/Labrasol ALF/PEG-400 in 150/478.1/159.4/212.5 volume ratio) for RLX-SMEDDS with a small droplet size (147.1 nm) and stable microemulsification (PDI: 0.227). Differential scanning calorimetry and powder X-ray diffraction of lyophilized RLX-SMEDDS revealed the loss of crystallinity, suggesting a molecularly dissolved or amorphous state of RLX in the SMEDDS formulation. Moreover, RLX-SMEDDS exhibited significantly higher saturation solubility and dissolution rate in water, simulated gastric fluid (pH 1.2), and simulated intestinal fluid (pH 6.8) than RLX powder. Additionally, oral administration of RLX-SMEDDS to female rats resulted in 1.94- and 1.80-fold higher area under the curve and maximum plasma concentration, respectively, than the RLX dispersion. Collectively, our findings suggest SMEDDS is a promising oral formulation to enhance the therapeutic efficacy of RLX.

## 1. Introduction

Raloxifene hydrochloride (RLX) is an effective drug used to protect postmenopausal women from breast cancer and osteoporosis by functioning as an estrogen antagonist and agonist in the breast and bone tissues, respectively [1]. Postmenopausal women are at a particularly high risk of developing osteoporosis and breast cancer, which increase their morbidity and mortality rates [2]. Hormone replacement therapy is often recommended to increase estrogen levels and relieve associated conditions, such as osteoporosis, in postmenopausal women [3]. However, undesirable side effects of long-term hormone replacement are a major reason for treatment discontinuation among patients [4]. Owing to its superior tissue selectivity with few side effects, RLX is a better choice than tamoxifen and other first-generation selective estrogen receptor modulators for postmenopausal women [1]. The recommended dose of RLX for the prevention of postmenopausal osteoporosis is 60 mg/day given orally in clinical use [5]. RLX exhibits a very low oral bioavailability of <2% in humans [6] and approximately 39% in rats [7]. Phase II glucuronide conjugation in the intestine is a major pathway for RLX metabolism, and raloxifene 6-β-D-glucuronide and 4′-β-D-glucuronide are the main metabolites in rats and humans, respectively [8,9].

Poor aqueous solubility of newly developed small drug molecules increases the difficulty of their formulation design to achieve high bioavailability after oral administration. More than 90% of the new US Food and Drug Administration-approved drugs have low or pH-dependent water solubility, leading to unpredictable absorption and suboptimal therapeutic efficacy [10]. Notably, 70% of these compounds are classified as Biopharmaceutics Classification System (BCS) class II drugs, and only 20% are classified as BCS class IV drugs [11,12]. RLX is a BCS class II drug, and hence, shows poor oral bioavailability (<2%) because of very low aqueous solubility (0.25 µg/mL) and extensive intestinal glucuronide conjugation [13,14]. Improving the solubility and dissolution rate of RLX will overcome its poor oral absorption. Various formulations, including lecithin–chitosan nanoparticles [15], solid dispersion [16,17], inclusion complexes [18], micro- and nano-emulsions [14,19], nanostructured lipid carriers [20,21], dry suspensions [22], mesoporous silica nanoparticles [23], phospholipid complexes [24], and polymeric nanoparticles [25,26], have been investigated to enhance the solubility and oral bioavailability of RLX. However, oral absorption of RLX remains low and continued efforts are required to improve its oral bioavailability. Lipid-based emulsion systems, such as the self-microemulsifying drug delivery system (SMEDDS), are used to improve the solubility, dissolution, and oral absorption of RLX due to their enormous solubilizing capacity and small droplet size with high surface area.

SMEDDS are clear and homogenous mixtures of drugs, oils, surfactants, and sometimes cosurfactants and cosolvents. Upon mild agitation with an aqueous medium in the gastrointestinal tract, this mixture produces a stable oil-in-water microemulsion after oral administration [27]. Use of two or more surfactants and cosurfactants greatly reduces the interfacial tension, and the presence of the drug in a solubilized form, accompanied by the small droplet size of SMEDDS enhances the dissolution profile and oral bioavailability of hydrophobic drugs [28]. SMEDDS can be administered in a liquid form using a soft gelatin capsule or in a powder form that is subsequently converted into tablets or filled in hard gelatin capsules [29]. Considering the advantages and huge potential of SMEDDS, we developed and optimized RLX-incorporated SMEDDS (RLX-SMEDDS) to enhance the solubility and oral bioavailability of RLX in this study. We also determined the optimal composition of RLX-SMEDDS by screening different vehicles based on drug solubility and constructing pseudo-ternary phase diagrams. The physicochemical properties and solid-state characteristics of optimized RLX-SMEDDS were also evaluated. The saturation solubility and dissolution profiles of lyophilized RLX-SMEDDS were examined in vitro using water, simulated gastric fluid (SGF; pH 1.2), and simulated intestinal fluid (SIF; pH 6.8) as the dissolution media. Additionally, oral bioavailability of RLX-SMEDDS was assessed in female Sprague–Dawley rats, and its pharmacokinetic parameters were compared with those of RLX dispersion.

## 2. Materials and Methods

### 2.1. Materials

RLX was purchased from Tokyo Chemical Industry Co., Ltd. (Tokyo, Japan). Labrafac lipophile WL 1349, Maisine CC, Capryol 90, and Labrasol ALF were gifted by Gattefossé (Saint-Priest, France). D-mannitol, linseed oil, isopropyl myristate, oleic acid, transcutol P, and polyethylene glycol 400 (PEG-400) were purchased from Daejung Chemical & Metal Co., Ltd. (Siheung, Republic of Korea). Tween 80 and Triton X-100 were purchased from Sigma-Aldrich (St. Louis, MO, USA). All other chemicals were of high-performance liquid chromatography (HPLC) or analytical grade.

### 2.2. Determination of RLX Solubility in Different Oils, Surfactants, and Cosurfactants

Solubilities of RLX in different oils, surfactants, and cosurfactants were measured according to a previously described protocol with minor modifications [30]. Briefly, 5 mL of each vehicle was placed in a borosilicate glass test tube and an excessive amount of RLX was added. The mixture was briefly sonicated, stirred, placed in a shaking water bath at 25 °C for 72 h, and oscillated at a rate of 100 oscillations/min. After achieving equilibrium, the mixture was centrifuged at 3000× *g* for 20 min and a clear solution in the supernatant was passed through a 0.22-µm polyvinylidene fluoride (PVDF) syringe filter, and the RLX content in each sample was estimated via HPLC analysis.

### 2.3. HPLC Analysis

Samples obtained from the solubility study were analyzed using the Agilent 1200 HPLC system (Agilent Technologies, Santa Clara, CA, USA) equipped with a G1322A degasser, G1311A quaternary pump, G1314B VWD detector, and Accucore C_18_ reversed phase column having a particle size, length, and internal diameter of 2.6 µm, 150 mm, and 4.6 mm, respectively (Thermo Fisher Scientific Inc., Waltham, MA, USA). The mobile phase was a mixture of acetonitrile and water (40:60, *v*/*v*) with trimethylamine (0.2%), and the pH was adjusted to 4.0 with diluted phosphoric acid. The flow rate, injection volume, and UV detector wavelength were set to 1.0 mL/min, 20 µL, and 290 nm, respectively [31].

A series of standard solutions of RLX were also prepared in methanol to construct a calibration curve. The obtained calibration curve for RLX in the concentration range of 1.5625 to 100 µg/mL showed very good linearity with a R^2^ value of 0.9999. The calibration curve was also reproducible as confirmed by multiple runs during preliminary tests. 

### 2.4. Construction of Pseudo-Ternary Phase Diagrams

Pseudo-ternary phase diagrams of systems containing oils, surfactants, and cosurfactants were constructed to identify the self-emulsifying regions. Briefly, varying proportions of the selected oil, surfactants, and cosurfactants from the solubility study were mixed with mild stirring, and the transition points were recorded using the water titration method, as previously reported [32]. The volume proportions of surfactant to cosurfactant (Km) were 3:1, 2:1, 1:1, 1:2, and 1:3, and at the selected Km value, ratios of oil to the surfactant/cosurfactant mixture varied between 9:1 and 1:9 in increments of 10%. Double distilled water was added dropwise to a mixture of oil, surfactants, and cosurfactants and stirred magnetically at 25 °C and the volume of water consumed was noted at a point where phase change (transparency/turbidity) occurred. The volumes of all components were normalized to their relative volume percentages, and ternary phase diagrams were plotted using the SigmaPlot 10.0 software (Systat Software Inc., San Jose, CA, USA).

### 2.5. Preparation of Liquid and Solid RLX-SMEDDS

Based on the solubility data, liquid RLX-SMEDDS was prepared using Capryol 90 as the oil, Tween 80/Labrasol ALF in a 3:1 ratio as the surfactant mixture, and PEG-400 as the cosurfactant. RLX concentration was kept constant at 1 mg/mL for all formulations. Accurately measured quantities of Capryol 90, Tween 80, Labrasol ALF, and PEG-400 were mixed in a glass vial under constant stirring and heating at 50 °C for 15 min. RLX was added to the heated mixture and stirred for an additional 10 min to obtain liquid RLX-SMEDDS. The formulations were kept at 25 °C for 2 days and observed for any cloudiness or phase separation before particle size measurement. The optimized liquid RLX-SMEDDS formulation was selected based on particle size analysis and microemulsion formation.

To prepare solid RLX-SMEDDS, mannitol was used as a solid carrier during the lyophilization process. Briefly, 6 mL of the optimized liquid RLX-SMEDDS was dispersed in 30 mL of aqueous mannitol solution (10%, *w*/*v*) under constant stirring for 10 min. The resulting mixture was lyophilized using a Freeze Dryer (TFD5503; IlShin BioBase Co., Ltd., Yangju, Republic of Korea). The mixture was prefrozen at −40 °C and subsequently freeze-dried at −55 °C and a pressure of <50 mTorr. Lyophilized RLX-SMEDDS was stored in a refrigerator until further use.

### 2.6. Droplet Size Analysis and Self-Microemulsifying Behavior of Liquid RLX-SMEDDS

Droplet size and polydispersity index (PDI) of the liquid RLX-SMEDDS formulation were determined using a zetasizer machine (ZS 90; Malvern Instruments, Malvern, Worcestershire, UK). Briefly, 10 µL of liquid RLX-SMEDDS was diluted with 20 mL of deionized water, and measurements were performed at room temperature and a scattering angle of 90° using the dynamic light scattering principle. Furthermore, the efficiency of self-microemulsion was evaluated using a standard USP dissolution apparatus II. One mL of each SMEDDS formulation was added dropwise to 200 mL of purified water at 37 °C and a paddle rotating speed of 60 rpm. The mixture was visually assessed for the rate of emulsification and final appearance and graded as a microemulsion if rapidly formed (<1 min) with a clear or slightly bluish appearance [33]. To further confirm the self-emulsification behavior, the optical clarity of SMEDDS formulae was evaluated by using a UV-Visible spectrophotometer. Liquid SMEDDS formulations were diluted 100-fold with deionized water and light transmittance (%) was measured at a wavelength of 650 nm using deionized water as the blank [34,35].

### 2.7. Characterization of Lyophilized RLX-SMEDDS

#### 2.7.1. Morphological Analysis

Surface morphologies of lyophilized RLX-SMEDDS, RLX powder, and mannitol were examined using scanning electron microscopy (SEM SU5000; Hitachi, Tokyo, Japan). The powdered samples were fixed on a brass stub with double-sided adhesive tape, coated with platinum under a vacuum using a Hitachi ion sputter, and imaged at an accelerating voltage of 20 kV. 

#### 2.7.2. Differential Scanning Calorimetry (DSC)

Thermal properties of lyophilized RLX-SMEDDS and related individual components were examined using a differential scanning calorimeter (DSC Q20; TA Instruments, New Castle, DE, USA). The sample material (3–5 mg) was accurately weighed, placed in an aluminum pan, and sealed with an aluminum cap. The analysis was conducted under a nitrogen purge of 20 mL/min, heating rate of 10 °C/min, and a heating range of 0–300 °C.

#### 2.7.3. Powder X-ray Diffraction (PXRD)

The crystalline states of lyophilized RLX-SMEDDS and related individual components were evaluated using a powder X-ray diffractometer (D/MAX-2500/PC; Rigaku Corporation, Tokyo, Japan) with a Cu K-α radiation source. The samples were scanned over a 2θ range of 3–70° at a step size of 0.02°. Tube voltage and current were maintained at 40 kV and 40 mA, respectively.

#### 2.7.4. Fourier-Transform Infrared (FTIR) Spectroscopy

The molecular dynamics of lyophilized RLX-SMEDDS were studied using a Fourier-transform infrared spectrophotometer (Alpha II; Bruker, Billerica, MA, USA). The samples were loaded in the disc and spectra were measured over the range of 400–4000 cm^−1^. 

### 2.8. Saturation Solubility and In Vitro Dissolution Study of RLX-SMEDDS

Saturation solubilities of RLX powder and lyophilized RLX-SMEDDS were determined in 3 different media: water containing 0.1% Tween 80, SGF (pH 1.2), and SIF (pH 6.8) [36]. Excess RLX powder or lyophilized RLX-SMEDDS was added to the respective media (4 mL) and incubated in a shaking water bath at 100 rpm and 37 °C for 72 h. The samples were filtered through a 0.45-µm PVDF syringe filter and analyzed for RLX content using a UV-Visible spectrophotometer (V-530; JASCO Corporation, Tokyo, Japan) at a wavelength of 290 nm. 

An in vitro dissolution study of RLX powder and lyophilized RLX-SMEDDS was also performed in the abovementioned three dissolution media using the USP dissolution apparatus II (Galvano Scientific Co., Ltd., Lahore, Pakistan). RLX powder (6 mg), or lyophilized RLX-SMEDDS equivalent to 6 mg of RLX, was added to 500 mL of the respective dissolution medium and the apparatus was operated at 100 rpm and 37 ± 0.5 °C. At predetermined time intervals of 10, 20, 30, 50, 70, 90 and 110 min, a sample of 5 mL was taken from the medium, filtered through the 0.45-µm syringe filter, and analyzed for RLX concentration using a UV-visible spectrophotometer at a λ_max_ of 290 nm. The original volume of the dissolution medium was maintained by adding an equivalent volume of fresh dissolution medium after withdrawal of each sample. 

### 2.9. In Vivo Pharmacokinetic Study

#### 2.9.1. Animals

In vivo pharmacokinetics of RLX-SMEDDS were assessed in female Sprague–Dawley rats (age: 9–10-weeks-old; weight: 220 ± 20 g) purchased from Orient Bio (Seongnam, Republic of Korea). All animals were acclimatized to a standard laboratory environment (25 °C average temperature and 12/12 h light/dark cycle) for one week before the experiments and given access to food and water ad libitum. The experimental protocols were reviewed and approved by the Institutional Animal Care and Use Committee of Gachon University (approval no. GU1-2023-IA0018-00; approval date: 13 April 2023).

#### 2.9.2. Oral Dosing, Blood Sampling, and Plasma Collection

A pharmacokinetic study in rats was performed as previously described [37,38,39]. Rats were fasted overnight and randomly assigned to either the RLX dispersion or RLX-SMEDDS group (*n* = 4). Rats were anesthetized via an intraperitoneal injection of zoletil (Vibrac, Westlake, TX, USA) and rompun (Bayer AG, Leverkusen, Germany), followed by cannulation of the femoral artery using polyethylene tubes (Clay Adams, Parsippany, NJ, USA) for blood sampling. Rats were orally administered the RLX dispersion (5 mg/mL) in 0.5% methyl cellulose or freshly prepared liquid RLX-SMEDDS (concentrated to 5 mg/mL using dimethyl sulfoxide as a cosolvent) at a dose of 10 mg/kg. Blood samples (120 µL) were withdrawn from the femoral artery at 0, 15, 30, 60, 120, 240, 360, 480, and 1440 min, and immediately centrifuged at 14,000 rpm for 15 min at 4 °C. Finally, plasma in the supernatant was separated and stored at −20 °C until analysis.

#### 2.9.3. Sample Preparation, Liquid Chromatography-Tandem Mass Spectrometry (LC-MS/MS) Analysis and Pharmacokinetic Parameters

Pharmacokinetic plasma samples were quantified for RLX concentration using LC-MS/MS analysis. Plasma samples were prepared for analysis by using the protein precipitation or deproteinization technique as it offers a number of advantages such as simplicity, low cost, minimal sample loss, and feasibility of automation [39,40,41]. Briefly, 50 µL of the plasma sample was mixed with 100 µL of the internal standard solution (100 ng/mL of phenacetin in methanol) and vortexed for 1 min to achieve deproteinization and drug extraction. The samples were then centrifuged at 14,000 rpm for 15 min at 4 °C and the supernatant was transferred to an analytical glass vial for LC-MS/MS analysis. Similarly, a series of calibration standards in rat plasma were prepared by mixing 90 µL of blank rat plasma with 10 µL of standard stock solution in methanol to obtain final RLX concentrations of 2, 5, 10, 20, 50, 100, 200, and 500 ng/mL. Finally, 200 µL of the phenacetin solution as an internal standard (IS) was added, followed by processing similar to that mentioned for the plasma samples above.

The prepared plasma standards and samples were analyzed to determine the RLX concentration using an AB SCIEX Triple Quad 3500 (TQ3500) mass spectrometer (AB Sciex LLC, Framingham, MA, U.S.A) connected to an Agilent 1290 HPLC system (Agilent Technologies). Chromatographic separation was performed using a Synergi polar reverse-phase column (pore size 80 Å, particle size 4 µm, dimensions 150 × 2 mm; Phenomenex, Torrance, CA, USA) and isocratic elution with a mobile phase of acetonitrile and 0.1% aqueous formic acid solution (70:30, *v*/*v*) pumped at a flow rate of 0.2 mL/min. The autosampler and column were maintained at 4 and 25 °C, respectively, and the sample injection volume was 2 µL. The multiple reaction monitoring (MRM) method was used to operate the TQ3500 mass spectrometer in positive electrospray ionization mode. MRM and instrument conditions were optimized to achieve maximum sensitivities for RLX and IS. The optimized MRM conditions and MS parameters for RLX were as follows: *m*/*z* of precursor ion: 474.024, *m*/*z* of product ion: 84.100, declustering potential: 136 V, entrance potential: 10 V, collision energy: 95 V, collision cell exit potential: 8 V, curtain gas pressure: 25 psi, collision gas pressure: 9 psi, ion spray voltage: 5500 V, temperature of ion source: 500 °C, nebulizing gas (GS1) pressure: 50 psi, and drying gas (GS2) pressure: 50 psi. Similarly, optimal MS parameters for IS (phenacetin) were as follows: *m*/*z* of precursor ion: 180.035, *m*/*z* of product ion: 110.0, declustering potential: 76 V, entrance potential: 10 V, collision energy: 27 V, collision cell exit potential: 8 V, curtain gas pressure: 25 psi, collision gas pressure: 9 psi, ion spray voltage: 5500 V, temperature of ion source: 500 °C, nebulizing gas (GS1) pressure: 50 psi, and drying gas (GS2) pressure: 50 psi. Analyst software (version 1.7.2, AB Sciex LLC, Framingham, MA, USA) was used for instrument control, data acquisition, and analysis. The validation of the LC-MS/MS method is described in detail in the Supplementary Material. 

Finally, the pharmacokinetic parameters were calculated from plasma drug concentration data using standard noncompartmental analysis with WinNonlin^®^ software version 8.3 (Pharsight Corporation, Mountain View, CA, USA).

### 2.10. Statistical Analysis

Data are represented as the mean ± standard deviation. Data were analyzed using unpaired Student’s *t*-test or one-way analysis of variance using the GraphPad Prism software (version 8.4.2; GraphPad Software, Inc. San Diego, CA, USA). Statistical significance was set at *p* < 0.05.

## 3. Results and Discussion

### 3.1. Selection of Oil, Surfactants, and Cosurfactants

Selection of appropriate components is of prime importance for the formation of clear, homogeneous, and stable microemulsions. Solubility is a major parameter in the screening of oils, surfactants, and cosurfactants, as it affects the solubilization capacity of poorly water-soluble drugs in SMEDDS [42]. RLX solubility was assessed in different oils, namely Capryol 90, isopropyl myristate, Labrafac lipophile WL 1349, and linseed oil, and oleic acid. Labrasol ALF, Maisine CC, Triton X-100, and Tween 80 were screened as surfactants, whereas PEG-400 and Transcutol P were evaluated as cosurfactants. The solubilities of RLX in different vehicles are presented in Table 1. Among the various medium- and long-chain fatty acid oils, RLX showed the highest solubility in Capryol 90 (259.9 µg/mL) and was therefore chosen as the oil phase. Likewise, RLX solubility was highest in Tween 80 (3195.1 µg/mL) followed by that in Labrasol ALF (246.4 µg/mL), and both of them were selected to form a surfactant mixture for RLX-SMEDDS formulations. Finally, PEG-400 was selected as the cosurfactant because of its higher RLX solubility (2950.3 µg/mL) than that of the other cosurfactants.

Medium-chain triglycerides are the preferred choice for lipid-based products because of their high affinity for lipophilic drugs, emulsification properties, and lack of susceptibility to oxidation during long-term storage [43]. Medium-chain triglycerides consisting of mono-, di-, and triglycerides are commonly used in microemulsions and SMEDDS formulations to enhance the oral absorption of lipophilic drugs [44,45]. SMEDDS form a stable oil-water emulsion with minimal agitation upon addition to water, as the surfactant and cosurfactant form an interfacial film, reduce the interfacial energy, and improve the thermodynamic stability by preventing coalescence [46]. Nonionic surfactants are generally considered safe and acceptable for oral formulations and have been used in several marketed formulations. Nonionic surfactants pose fewer toxicity concerns than cationic and anionic surfactants, and bulkier surfactants, such as polysorbates, are safer than single-chain surfactants [43]. The Griffin’s hydrophilic-lipophilic balance (HLB) scale, designed specifically for nonionic surfactants, ranges between 0 and 20. The HLB system has now been extended to ionic surfactants having much higher HLB values of up to 45, based on their ionization properties. Typically, nonionic surfactants are preferred over ionic surfactants for developing drug formulations as they provide greater resistance to changes in pH or ionic strength. Emulsifiers with HLB values of 3–8 and 8–18 were used to form water-in-oil and oil-in-water microemulsions, respectively. Moreover, stable microemulsions are often formed when a combination of surfactants with different HLB values are used together [47].

A mixture of nonionic surfactants, Tween 80/Labrasol ALF (3:1), was used to prepare RLX-SMEDDS in the present study. Tween 80 and Labrasol ALF are nonionic emulsifiers with HLB values of 15 and 12, respectively [48]. The combined use of nonionic surfactants resulted in smaller droplet sizes, shorter emulsification times, and better stability of SMEDDS formulations [49,50]. A ratio of 3:1 was selected for the Tween 80/Labrasol ALF mixture because of its higher HLB value of 14.25 compared to the other proportions (2:1, 1:1, 1:2, and 1:3), with HLB values of 14.01, 13.5, 12.99, and 12.75, respectively. Cosurfactants are amphiphilic molecules that increase the flexibility of interfacial films by accumulating at the interface alongside surfactant molecules, thus allowing the formation of stable microemulsions under various conditions and compositions [32]. The use of a hydrophilic cosurfactant with an HLB value of 10–14 is often preferred in SMEDDS formulations for spontaneous microemulsion formation [46]. Therefore, we used PEG-400 with an HLB value of 12 for RLX-SMEDDS. It is also important to consider the total intake of oils and surfactants for safety when designing oral formulations. All the ingredients (oil, surfactants, and cosurfactants) used in our formulation are of the generally recognized as safe (GRAS) category. More importantly, we calculated the dose volume of oil and surfactants by considering the volume (2 mL/kg) of RLX-SMEDDS formulation given to rats during the pharmacokinetics study. The intake of Capryol 90, Tween 80, Labrasol ALF, and PEG-400 was 0.27, 0.86, 0.29, and 0.38 mL/kg, respectively, in our study. These doses are well below the maximum tolerated levels of these ingredients (for rodents; Oils: 5 mL/kg, Tween 80: 1.25 mL/kg, Labrasol ALF: 2 mL/kg, PEG-400: 2 mL/kg) reported in preclinical studies and discussed in detail elsewhere [51,52]. Overall, the selected ingredients were well tolerated and widely used in pharmaceutical formulations.

### 3.2. Pseudo-Ternary Phase Diagrams

**Figure 1:**
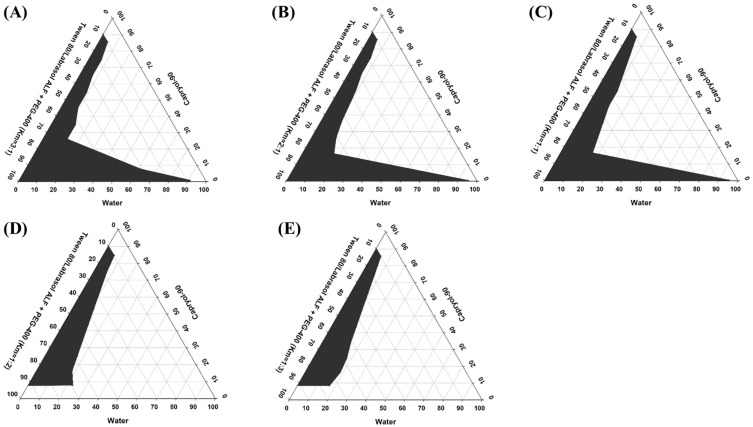
Pseudo-ternary phase diagrams of the self-microemulsifying drug delivery system (SMEDDS) prepared using Caproyl-90 as an oil, Tween 80/Labrasol ALF as a surfactant mixture, and polyethylene glycol (PEG)-400 as a cosurfactant. Different surfactant to cosurfactant ratios (Km) of 3:1 (**A**), 2:1 (**B**), 1:1 (**C**), 1:2 (**D**), and 1:3 (**E**) were investigated. Gray regions in pseudo-ternary phase diagrams indicate the microemulsion phase.

Pseudo-ternary phase diagrams were constructed to determine the appropriate concentration range for each component of SMEDDS. An ideal vehicle should have good solubilizing capacity for the drug and a large self-microemulsifying region in its ternary phase diagram [53,54]. A series of blank SMEDDS were prepared with varying ratios of oil (Capryol 90), surfactant (Tween 80/Labrasol ALF mixture), and cosurfactant (PEG-400) selected from the solubility study, and their corresponding ternary phase diagrams were constructed, as shown in Figure 1. Five different systems with surfactant/cosurfactant ratios (Km) of 3:1, 2:1, 1:1, 1:2, and 1:3 were tested. Among the tested compositions, the constructed pseudo-ternary phase diagrams exhibited the largest self-microemulsifying region with a Km ratio of 3:1 (Figure 1A). Upon increasing the proportion of the cosurfactant (2:1, 1:1, 1:2, and 1:3), a gradual decrease in the self-microemulsifying region, and thus, poor emulsion-forming ability, was observed (Figure 1B–E). Increasing the cosurfactant (PEG-400) concentration in SMEDDS decreased the net HLB value of the system, resulting in a lower self-microemulsifying area. A similar correlation between Km ratios, HLB values, and emulsification areas has been reported elsewhere [48]. Based on these results, a Tween 80/Labrasol ALF to PEG-400 ratio of 3:1 was selected for further optimization and development of RLX-SMEDDS.

### 3.3. Optimization of RLX-SMEDDS

**Figure 2:**
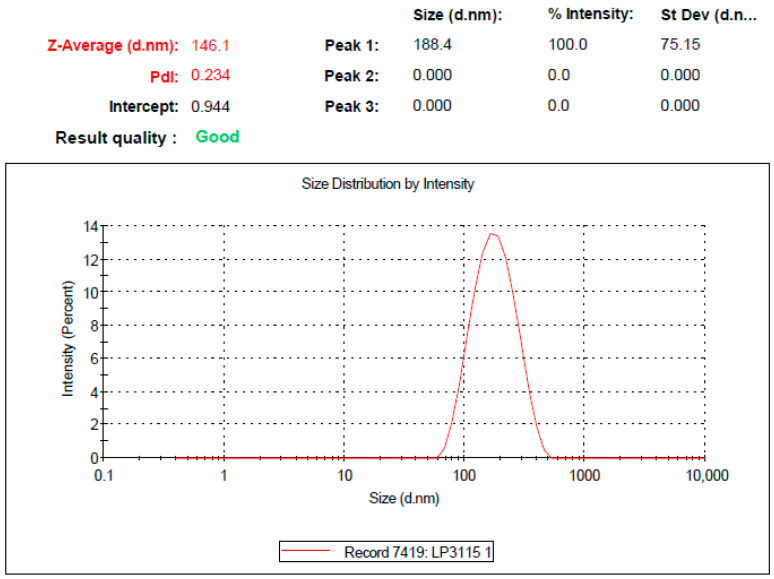
Droplet size distribution curve of the optimized formulation of liquid RLX-incorporated SMEDDS (RLX-SMEDDS) measured using the dynamic light scattering (DLS) technique after 100-times dilution with double distilled water.

For the selected Km value of 3:1, different formulations were prepared with varying ratios of oil-to-surfactant/cosurfactant mixtures in the range 1:9–9:1. The droplet size and microemulsion formation behavior for different combinations of oil and surfactant/cosurfactant mixtures at Km = 3:1 are presented in Table 2. The results indicated that the droplet size significantly increased when the surfactant/cosurfactant concentration was decreased in the RLX-SMEDDS formulations (F1 to F6), with a few exceptions (F7 and F8). The dispersed phase of a microemulsion is composed of small droplets of oil surrounded by surfactant and cosurfactant molecules at the oil/water interface [55]. At high surfactant concentrations, the interfacial film is stabilized and compressed, resulting in a decrease in the particle size [32]. Additionally, the extremely small particle size at low oil concentrations (F1) may be attributed to the formation of micelles instead of emulsions [56]. In contrast, the low surfactant concentrations in F9 and F10 resulted in inadequate emulsification with a considerably large droplet size. It should be noted that oil to surfactant/cosurfactant mixture ratios were initially increased by 10%. However, the F1 formulation (10:90) did not form a microemulsion, whereas the F3 formulation (20:80) showed a particle size >200 nm with a very large PDI. Therefore, a new formulation (F2) was designed with an oil-to-surfactant/cosurfactant mixture ratio of 15:85. RLX-SMEDDS formulation F2 composed of Capryol 90, Tween 80, Labrasol ALF, and PEG-400 in 150/478.1/159.4/212.5 volume ratios showed the optimal droplet size and PDI of 147.1 ± 1.0 nm and 0.227 ± 0.01, respectively. The low PDI value was also accompanied by a unimodal and narrow particle size distribution curve (Figure 2), which showed a homogeneous distribution of droplets in the RLX-SMEDDS formulation. In addition, the optimized RLX-SMEDDS formulation showed a particle size and PDI of 119.5 ± 4.6 nm and 0.193 ± 0.029 in a physiological buffer of pH 1.2, respectively. Similarly, the droplet size and PDI of RLX-SMEDDS were 99.9 ± 4.1 nm and 0.185 ± 0.012 in a buffer of pH 6.8. The slightly lower particle sizes in physiological buffers than in deionized water might have favorable effects on absorption in the GI tract. It is noteworthy that the droplet size of microemulsions is an important factor in their oral absorption, as a smaller droplet size results in an increased surface area and, thereby, better absorption [57,58]. In addition, a droplet size of less than 200 nm is a characteristic feature of SMEDDS [28]. Optical clarity is also an important feature to describe and differentiate microemulsions from ordinary emulsions. Emulsions are optically turbid (cloudy) whereas SMEDDS are optically clear (transparent) upon dilution [28,59]. Table 2 presents the percentage of transmittance for F1 to F10 formulations. High transmittance corresponds to optical clarity and vice versa. F3 to F5 formulations were clear on visual observation, however they exhibited transmittance of less than 80% upon optical clarity indicating their tendency to form ordinary emulsions. On the other hand, the F2 formulation was clear on visual observation and showed transmittance of 96.8% which shows it remained as microemulsion upon dilution. Percentage of transmittance can also be directly correlated to droplet size as large droplets scatter most of the incident light with a resultant low transmittance [34]. Taken together, F2 was selected as a stable optimized formulation based on droplet size, PDI, microemulsifying behavior, and optical clarity. It is pertinent to mention the overlapping similarities between SMEDDS and self-nanoemulsifying drug delivery systems (SNEDDS), which lead to considerable confusion in the literature about their precise nature and characteristics. The droplet size ranges for SMEDDS and SNEDDS are reported differently in the literature; however, both of these formulations are less than 250 nm [28,60,61]. On the basis of classification proposed for lipid-based solutions, SNEDDS and SMEDDS are classified as type IIIA and IIIB lipid formulations, respectively [62,63]. According to this classification, SNEDDS are composed of higher oil proportions (40–80%), whereas SMEDDS usually contain less than 20% oil with high proportions of hydrophilic surfactants (20–50%) and cosurfactants (20–50%). Furthermore, nanoemulsions are thermodynamically unstable systems, whereas microemulsions are thermodynamically stable. Microemulsions are formed with mild agitation of oil, water, and surfactants without any external energy, whereas nanoemulsions always require the input of some external energy (high-pressure homogenization, ultrasonication, etc.) to convert separate ingredients into a colloidal dispersion [64]. RLX-SMEDDS in our study were composed of less than 20% oil, involved only mild heating and stirring for their preparation, and a water titration method to construct pseudo ternary phase diagrams for identifying the self-microemulsifying regions. Collectively, particle size within the stated range, composition similar to type IIIB lipid formulation, preparation without external energy, emulsification behavior, and stability (evidenced by the absence of any phase separation throughout the course of the study) demonstrated that a microemulsion rather than a nanoemulsion was formed in our study. Therefore, SMEDDS rather than SNEDDS accurately describes the nature of our formulation. 

**Figure 3:**
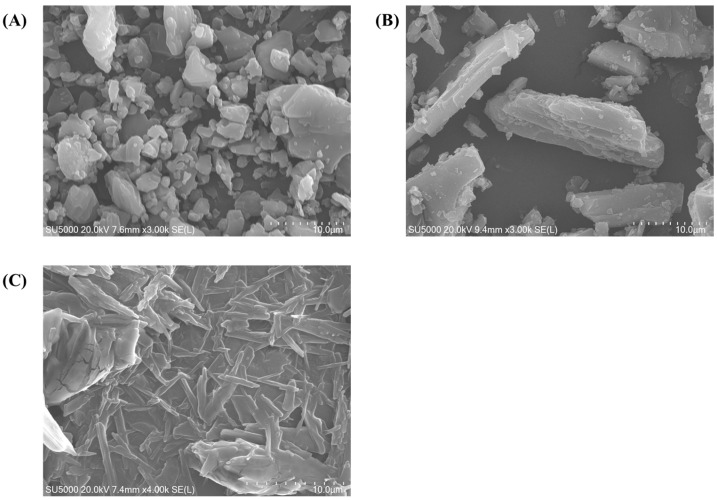
Scanning electron microscopy (SEM) micrographs of RLX (**A**), mannitol (**B**), and lyophilized RLX-SMEDDS (**C**).

Surface morphologies of RLX powder, mannitol, and lyophilized RLX-SMEDDS are shown in the SEM images in Figure 3. RLX exhibited a distinct crystalline morphology with irregularly shaped crystals, whereas mannitol exhibited a needle-like elongated morphology. In contrast, lyophilized RLX-SMEDDS demonstrated a relatively smooth surface and matrix-like morphology with no clear crystalline appearance of RLX, suggesting complete dissolution and perfect incorporation into SMEDDS.

### 3.4. Solid State Characteristics of RLX-SMEDDS

**Figure 4:**
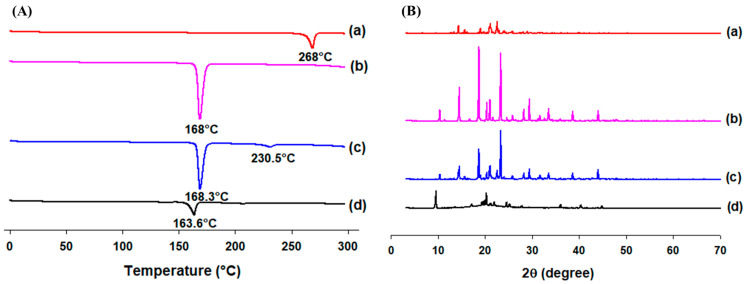
Differential scanning calorimetry (DSC) thermograms (**A**) and powder X-ray diffraction (PXRD) patterns (**B**) of RLX (a), mannitol (b), physical mixture (c), and lyophilized RLX-SMEDDS (d).

Thermal behaviors of RLX powder, mannitol, the physical mixture, and lyophilized RLX-SMEDDS are shown in the DSC thermograms in Figure 4A. RLX powder and mannitol demonstrated sharp endothermic peaks at about 268 and 168 °C, respectively, which correspond to their respective melting points [17]. The physical mixture of RLX and mannitol (1:1) showed a less prominent and broadened endothermic peak for RLX at 230.5 °C and a sharp peak for mannitol at 168.3 °C. This slight shift in the melting endotherm of RLX in the physical mixture could be attributed to its partial dissolution in molten mannitol, thereby lowering its crystallinity and resulting in a peak of lower intensity at a slightly lower temperature [65]. In contrast, the endothermic peak for RLX completely disappeared in the DSC thermogram of lyophilized RLX-SMEDDS, indicating its complete dissolution in the formulation and transformation from the original crystalline to an amorphous form. The endothermic melting peak at a slightly lower temperature (163.6°C) still exists for mannitol in the thermogram of RLX-SMEDDS.

The molecular dissolution state of RLX in SMEDDS was further confirmed by examining its crystallinity using PXRD diffractograms (Figure 4B). RLX powder exhibited several distinctive diffraction peaks at 14.36°, 15.68°, 19.04°, 21.10°, and 22.60°, indicating a crystalline state. These crystalline peaks were similar to those reported elsewhere [16]. Similarly, the PXRD pattern of mannitol displayed intense peaks at 14.54°, 18.7°, 20.4°, 21.06°, 23.32°, and 29.4°, demonstrating its crystalline characteristics. The physical mixture showed diffraction peaks for RLX and mannitol, demonstrating that both compounds retained their crystallinity during PXRD, unlike in the DSC analysis, where some portion of RLX was dissolved in mannitol during the heating process, and its crystallinity was reduced. Moreover, most of the sharp characteristic peaks of RLX disappeared in lyophilized RLX-SMEDDS, and the majority of the small diffractions seen in PXRD of lyophilized RLX-SMEDDS could be ascribed to the presence of mannitol in lyophilized RLX-SMEDDS. The findings suggest the conversion of RLX to a molecularly dissolved or amorphous state within the SMEDDS matrix, in agreement with the DSC results.

**Figure 5:**
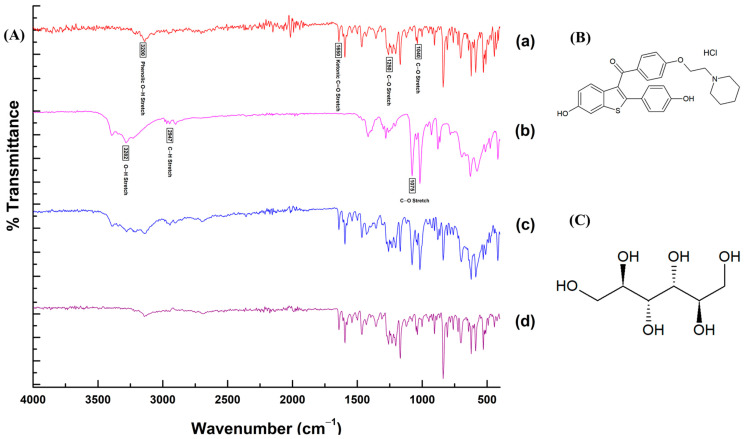
Fourier-transform infrared (FTI)R spectra (**A**) of RLX powder (a), mannitol (b), physical mixture (c), and lyophilized RLX-SMEDDS (d), and chemical structures of RLX (**B**) and mannitol (**C**).

The results of the FTIR analysis demonstrating the molecular interactions of RLX with other ingredients in the SMEDDS formulation are presented with the FTIR spectra and chemical structures in Figure 5. The characteristic functional groups of RLX showed sharp peaks corresponding to the ketonic C=O stretching vibration at 1650 cm^−1^, phenolic O–H stretching at 3200 cm^−1^, and the C–O stretching of phenyl ethyl ether at 1250 and 1040 cm^−1^. Most of the characteristic bands of RLX and mannitol were present in the FTIR spectra of the mixture as well as RLX-SMEDDS. The absence of any major shift in bands or the appearance of no new bands in the FTIR spectra of RLX-SMEDDS demonstrated that there was no chemical interaction or bond formation of RLX with other ingredients. However, the dissolution of crystalline RLX in SMEDDS may have resulted in a slight reduction in the peak intensities of the ketonic and phenolic groups. Overall, the DSC, PXRD, and FTIR analyses’ results were in agreement with each other.

### 3.5. Saturation Solubility and Dissolution Profile of RLX-SMEDDS

**Figure 6:**
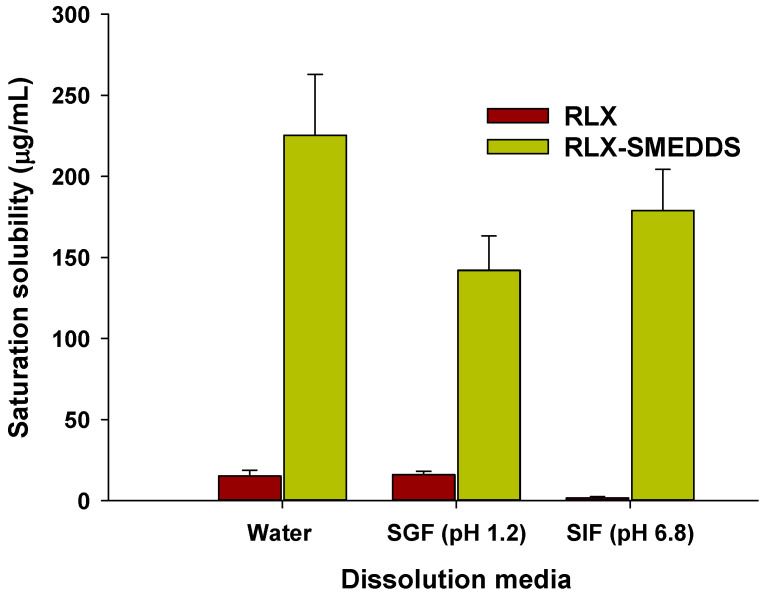
Saturation solubilities of RLX and lyophilized RLX-SMEDDS in water, simulated gastric fluid (SGF; pH 1.2), and simulated intestinal fluid (SIF; pH 6.8). Data are represented as the mean ± S.D. (*n* = 3).

Saturation solubilities of RLX powder and lyophilized RLX-SMEDDS in different physiological media are presented in Figure 6. Moreover, the pH-solubility profile of RLX is shown in the Supplementary Material Figure S1. At equilibrium, the saturation solubility of RLX-SMEDDS in water (225.3 vs. 15.2 µg/mL), SGF (142.2 vs. 16.0 µg/mL), and SIF (178.9 vs. 1.7 µg/mL) was substantially higher than that of RLX powder (Figure 6). These results indicate that RLX-SMEDDS significantly improved the saturation solubility of RLX by 14.8-, 8.9-, and 105-fold increases in water, SGF, and SIF, respectively. The increased saturation solubility of RLX-SMEDDS can be ascribed to the smaller particle size and amorphous nature of RLX within the SMEDDS formulation. These factors collectively enhance the partitioning of the drug into lipid droplets and potential micelles, leading to improved saturation solubility [62,66].

**Figure 7:**
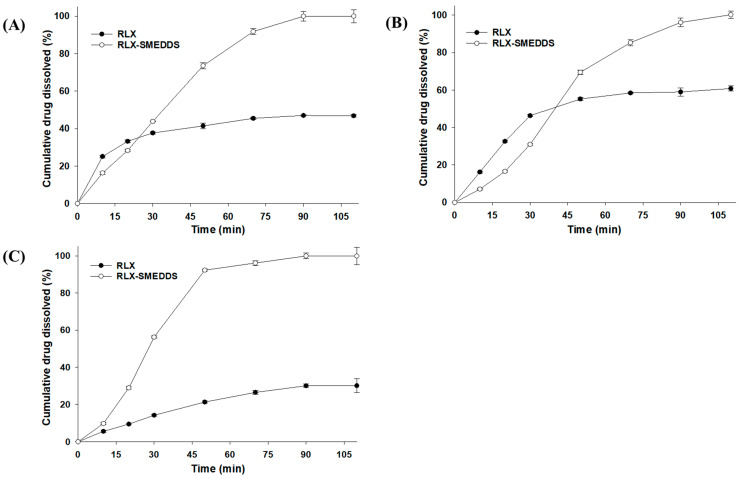
Dissolution profiles of RLX and lyophilized RLX-SMEDDS in water (**A**), simulated gastric fluid (pH 1.2) (**B**), and simulated intestinal fluid (pH 6.8) (**C**). Data are represented as the mean ± S.D. (*n* = 3).

In vitro dissolution profiles of lyophilized RLX-SMEDDS and RLX powder in different dissolution media are shown in Figure 7. RLX powder had a noticeably low dissolution rate owing to its poor solubility in media. Approximately 30–60% of the RLX powder was dissolved in 110 min, and the cumulative drug dissolution was in the order of SGF (60.8%) > water (46.9%) > SIF (30.2%). The dissolution profiles of RLX powder were consistent with the saturation solubility results, and the higher dissolution rate of RLX in SGF could be ascribed to its higher solubility than that in SIF [67]. In contrast, the RLX-SMEDDS showed substantially higher dissolution rates than RLX powder in each dissolution medium. From Figure 7, it is evident that approximately 80% of RLX was dissolved from RLX-SMEDDS in the first 60 min in all 3 media. These enhanced RLX dissolution velocities could be ascribed to the fact that completely dissolved RLX in SMEDDS was quickly released into the medium by the spontaneous formation of small droplets [27]. The collective contributions from the molecularly dissolved/amorphous state of the drug, improved saturation solubility, small droplet size, and low interfacial energy resulted in the higher dissolution rate of RLX-SMEDDS compared to that of RLX powder. 

### 3.6. Pharmacokinetics Profile 

A simple, reliable, and sensitive LC-MS/MS method was developed to determine RLX concentrations in rat plasma samples. The optimized MRM and chromatographic conditions resulted in efficient peak resolution, with sharp peaks for RLX and phenacetin at 3.37 and 2.35 min, respectively. These retention times for RLX and the IS remained almost the same for all standard and plasma samples which demonstrate adequate selectivity of the method. The calibration curve for RLX in rat plasma constructed in the concentration range of 2–500 ng/mL showed very good linearity, as demonstrated by a high coefficient of correlation (r = 0.9992 with a weighing factor of 1/x). The calibration curve of RLX is presented in the Supplementary Material Figure S2. The accuracy for all the standard concentrations was between 89.9% and 110%. These results indicate that response (RLX to IS peak area ratio) is directly proportional to the RLX to IS concentration ratio in plasma samples, thus showing linearity of the method.

The intraday accuracy and precision at four different QC concentrations of RLX in rat plasma is shown in the Supplementary Material Table S1. The intraday accuracy for RLX was in the range of 95.17–107.58% with absolute %RE of 0.39–7.58%. Similarly, the intraday precision for RLX in QC samples was found to be ≤3.171%. The accuracy and precision data for RLX was well within the acceptable limits of ≤20% for LLOQ and ≤15% for all other QC samples, as specified by the US-FDA guidelines for bioanalytical methods validation [68]. 

The recovery, extraction efficiency, and matrix effects were also evaluated at four different QC concentrations of RLX using five different sources of rat plasma and the results are shown in the Supplementary Material Table S2. As shown in the results, total recovery and extraction efficiency ranged between 90.75% and 99.25% and 94.76% and 96.35%, respectively, for all QC samples. These finding suggests the suitability of the protein precipitation method to adequately extract RLX from rat plasma. Furthermore, the absolute matrix effect was also similar for all QC levels (94.28–103.09%) in all plasma sources. The relative matrix effect, which shows the variability in peak areas of RLX spiked in extracted plasma samples in the same concentration level, was also comparable between different QC levels. The relative matrix effect ranged between 1.09% and 3.07% for all QC levels, which indicates the absence of a significant effect from the plasma matrix for the analysis of RLX in rat plasma. Taken together, the results of method validation demonstrated the adequacy and suitability of the developed LC-MS/MS method.

**Figure 8:**
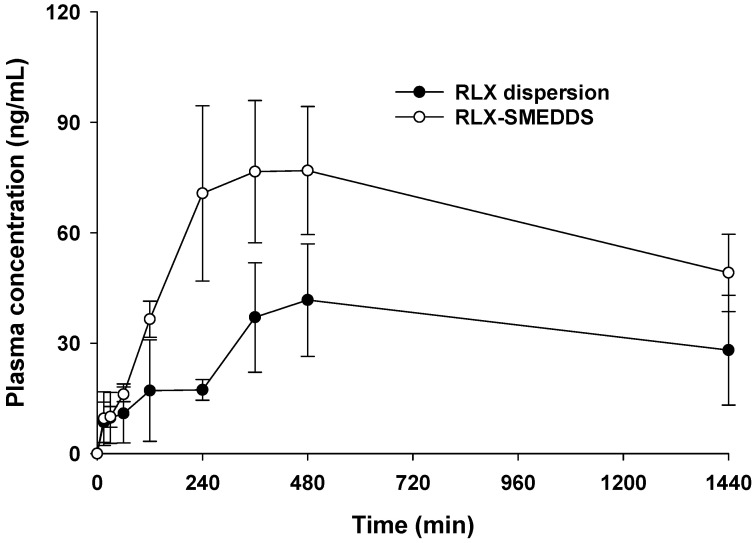
Average RLX plasma concentration–time profiles after the oral administration of RLX dispersion and RLX-SMEDDS to female rats at a dose equivalent to 10 mg/kg. Data are represented as the mean ± S.D. (*n* = 4).

The in vivo pharmacokinetics study for RLX-SMEDDS was conducted in female rats at a dose of 10 mg/kg. This dose for rats was decided by converting the daily recommended dose of RLX in human (60 mg daily, or 1 mg/kg assuming average human body weight of 60 kg) to animal equivalent dose. According to the US-FDA guidance, the interconversion of human and animal equivalent doses are based on the concept of conversion factor calculated by normalization of dose to body surface area [69,70]. By using human to animal dose conversion, rat equivalent dose was calculated as 6.2 mg/kg. Based on these calculations and a pilot pharmacokinetics study, we used a slightly higher dose of 10 mg/kg for pharmacokinetics study in rats. Plasma drug concentration vs. time profiles of RLX-SMEDDS and RLX dispersion after oral administration to female rats are shown in Figure 8. RLX-SMEDDS exhibited a higher plasma drug concentration than RLX dispersion, and the difference in plasma concentration was more prominent after 120 min. The noncompartmental pharmacokinetics parameters calculated from the plasma RLX concentration vs. time data are listed in Table 3. RLX-SMEDDS exhibited significantly higher area under the concentration-time curve from 0 to 24 h (AUC_24h_; 87,144.5 vs. 44,907.5 ng × min/mL; *p* < 0.01) and maximum concentration (C_max_; 81.6 vs. 45.6 ng/mL; *p* < 0.05) than RLX dispersion. Both AUC_24h_ and C_max_ are key indicators for describing and comparing the oral bioavailability of formulations. Furthermore, the relative bioavailability (BA_rel_) of RLX-SMEDDS was 194% of that of the RLX dispersion. The enhanced absorption and oral bioavailability of RLX-SMEDDS may be attributed to the large surface area provided by tiny microemulsion droplets and improved diffusion, solubility, and dissolution in the gastrointestinal tract, and enhanced mucosal permeability due to surfactants [71]. Indeed, RLX dispersion exhibited poor and slow absorption owing to its very low solubility and slow dissolution rate in intestinal fluid, as evidenced by its saturation solubility (Figure 6) and dissolution profile (Figure 7). Although RLX-SMEDDS improved the oral bioavailability of RLX by almost two-fold, the time to reach the maximum drug concentration (T_max_) was long, and the overall plasma drug concentration was lower than that expected from the solubility and dissolution data. The slightly delayed absorption and long T_max_ of RLX-SMEDDS may be due to a very long elimination half-life of RLX and the possible contribution and involvement of the intestinal lymphatic pathway. RLX possesses an elimination half-life of 27–32 h in humans because of reversible systemic metabolism and significant enterohepatic cycling of the drug [72]. Since T_max_ is governed by the rates of drug absorption and elimination, slow absorption and elimination of drugs result in high T_max_ values. Slow lymphatic flow may also contribute to slow absorption and delayed T_max_ of RLX-SMEDDS; such findings with lymphatic absorption have also been previously reported [73,74]. In addition, presystemic glucuronide metabolism of RLX in the intestine is a major determining step for oral absorption [8] that may affect the pharmacokinetic parameters of RLX-SMEDDS. Finally, low water content (3.2 mL) in the gastrointestinal tract of fasted rats may also affect the self-microemulsifying process of SMEDDS, and thus, the oral absorption [75]. Previously, somewhat comparable pharmacokinetic results have been reported for RLX in studies intended to improve its oral bioavailability via nanostructured lipid carriers (NLCs). NLCs prepared from glyceryl tribehenate and oleic acid resulted in a 3.19-fold enhancement in oral bioavailability compared to RLX suspension at a dose of 15 mg/kg [20]. In another study, NLCs formulated with glyceryl monostearate and Capmul MCM C8 showed a 3.75-fold increase in oral bioavailability compared to RLX suspension administered at a dose of 15 mg/kg [21]. Despite variability in the relative oral bioavailability data between our RLX-SMEDDS and their RLX-NLCs formulations, RLX suspension showed similarity in terms of AUC_24h_, C_max_, T_max_ and MRT. Taken together, a 1.94-fold enhancement in oral bioavailability (BA_rel_, %) by RLX-SMEDDS demonstrates its usefulness for the oral delivery of RLX.

## 4. Conclusions

In this study, we successfully developed an SMEDDS formulation for the effective oral delivery of the poorly water-soluble drug, RLX. The formulation components and their proportional ratios were determined through a solubility study, construction of pseudo-ternary phase diagrams, droplet size, and emulsification ability measurements. The developed RLX-SMEDDS successfully enhanced the solubility and dissolution of RLX in different physiological media, namely water, SGF, and SIF. Furthermore, key pharmacokinetic parameters of RLX, such as AUC and C_max_, were also significantly improved by RLX-SMEDDS after oral administration to rats, indicating improved in vivo absorption compared to that of RLX powder. Therefore, SMEDDS is a promising formulation that can overcome the drawbacks associated with the poor solubility and oral bioavailability of RLX.

## Figures and Tables

**Table 1:** Solubilities of raloxifene hydrochloride (RLX) in different oils, surfactants, and cosurfactants.

Vehicle	Description and Composition	RLX Solubility (µg/mL)
**Oils**		
Capryol 90	Propylene glycol caprylate	259.9 ± 37.5
Labrafac lipophile WL 1349	Medium-chain triglycerides of caprylic (C8) and capric (C10) acids	156.1 ± 22.3
Linseed oil	Long chain fatty acid	148.1 ± 8.3
Isopropyl myristate	Isopropyl tetradecanoate	111.4 ± 33.0
Oleic acid	Long chain fatty acid	41.3 ± 16.6
**Surfactants**		
Tween 80	Polyoxyethylene sorbitan monooleate	3195.1 ± 86.8
Labrasol ALF	Caprylocaproyl macrogol-8 glycerides	246.4 ± 22.4
Maisine CC	Glyceryl monolinoleate	105.6 ± 22.1
Triton X-100	Polyoxyethylene octyl phenyl ether	41.5 ± 18.7
**Cosurfactants**		
Polyethylene glycol 400	Polyethylene glycol	2950.3 ± 73.5
Transcutol P	Diethylene glycol monoethyl ether	2079.5 ± 62.7

Data are represented as the mean ± standard deviation (S.D.). *n* = 3.

**Table 2:** Optimization of the RLX-incorporated self-microemulsifying drug delivery system (RLX-SMEDDS) based on droplet size and microemulsion formation.

Formulation	Capryol 90 (%)	Tween 80/Labrasol ALF/PEG-400 Mixture (%)	Droplet Size (nm)	PDI	Microemulsion Formed (Visual Inspection)	% Transmittance
F1	10	90	18.4 ± 0.1	0.208 ± 0.01	No	99.3 ± 0.57
F2	15	85	147.1 ± 1.0	0.227 ± 0.01	Yes	96.8 ± 0.03
F3	20	80	258.2 ± 8.2	0.555 ± 0.03	Yes	75.4 ± 1.19
F4	30	70	470.9 ± 22.3	0.494 ± 0.23	Yes	61.2 ± 0.2
F5	40	60	610.6 ± 51.4	0.526 ± 0.29	Yes	46.4 ± 0.07
F6	50	50	669.9 ± 56.9	0.499 ± 0.21	No	13.5 ± 0.07
F7	60	40	297.6 ± 2.8	0.419 ± 0.05	No	6.3 ± 0.09
F8	70	30	452.9 ± 20.2	0.298 ± 0.01	No	7.8 ± 0.07
F9	80	20	633.9 ± 38.7	0.331 ± 0.17	No	4.7 ± 0.13
F10	90	10	2792.0 ± 58.1	0.567 ± 0.43	No	6.1 ± 0.23

RLX concentration was 1 mg/mL for all formulations. Data are represented as the mean ± S.D. (*n* = 3).

**Table 3:** Non-compartmental analysis of the pharmacokinetic parameters after the oral administration of RLX dispersion and RLX-SMEDDS to female rats at a dose equivalent to 10 mg/kg of RLX.

Parameters	RLX Dispersion	RLX-SMEDDS
AUC_24h_ (ng × min/mL)	44,907.5 ± 15,657.7	87,144.5 ± 13,815.1 **
C_max_ (ng/mL)	45.6 ± 16.1	81.6 ± 17.0 *
T_max_ (min)	420.0 ± 69.3	390.0 ± 114.9
MRT (min)	712.4 ± 79.6	688.51 ± 71.5
BA_rel_ (%)	-	194.0

AUC_24h_, area under the plasma drug concentration-time curve from 0 to 24 h; C_max_, maximum plasma drug concentration; T_max_, time to reach maximum plasma drug concentration; MRT, mean residence time; BA_rel_: relative bioavailability. Data are represented as the mean ± S.D. (*n* = 4). * *p* < 0.05 and ** *p* < 0.01 vs. RLX dispersion.

## Data Availability

All data are included in this manuscript.

## Supplementary Materials

The following supporting information can be downloaded at: https://www.mdpi.com/article/10.3390/pharmaceutics15082073/s1. References [76,77] are cited in the Supplementary Materials. Figure S1. pH-solubility profile of RLX (*n* = 3). Figure S2. Calibration curve for RLX in the concentration range of 2–500 ng/mL). Table S1. Intraday accuracy and precision of the analytical method for the quantification of RLX in rat plasma. Table S2. Recovery, extraction efficiency and matrix effects for RLX in rat plasma.
