# Supplementary material for: Formulation and Evaluation of a Self-Microemulsifying Drug Delivery System of Raloxifene with Improved Solubility and Oral Bioavailability
Source: Pharmaceutics. 2023 Aug 2;15(8):2073. doi: 10.3390/pharmaceutics15082073 (PMC10459739; doi:10.3390/pharmaceutics15082073)
Supplement: Supplementary file 1 [file pharmaceutics-15-02073-s001.zip › pharmaceutics-2517783-supplementary.pdf]

__Supplementary Materials__

Formulation and Evaluation of a Self-Microemulsifying Drug Delivery System of Raloxifene with Improved Solubility and Oral Bioavailability

1. Measurement of pH-solubility profile of RLX

The pH-solubility profile of RLX was evaluated in different buffer solutions with pHs ranging between 1.2 and 8 to cover pHs of physiological relevance. The buffer solutions were prepared according to the composition specified in the USP 30 NF25. Buffer solutions with a pH of 1.2, 2.0, 4.5, 5.5, 6.8, 7.4, and 8 were prepared, and an excess amount of RLX was added to 4 mL of each buffer medium. Buffers with RLX added were incubated in a shaking water bath set at 37°C and 100 rpm for a period of 48 h. The samples were then centrifuged at 8500 ×g for 15 min, and a supernatant was collected and filtered through a 0.45-μm PVDF syringe filter to remove the undissolved RLX. The filtered samples were then subjected to RLX quantification using a UV-Visible spectrophotometer (V-530; JASCO Corporation, Tokyo, Japan) at a wavelength of 290 nm.

The solubility of RLX measured in different buffer solutions is presented in **Figure S1**. From the results, RLX showed the highest solubility in a buffer solution with a pH of 4.5 (68.75 ± 2.54 μg/mL) and the lowest solubility at a pH of 8 (0.13 ± 0.03). RLX is regarded as a zwitterion compound due to its possession of both acidic and basic functional groups [1]. RLX has phenolic hydroxyl groups to act as an acid whereas it also has the basic nitrogen. The presence of these functional groups allows RLX to display either acidic or basic properties depending on the pH of the medium, which in turn effects its solubility at different pHs. It has been reported that RLX possesses three p*K*a values at 8.95, 9.83 and 10.91 [1], suggesting that RLX would be increasingly insoluble as the pH increases to about 9. Its ionic behavior suggests that RLX would exhibit higher solubility in acidic buffers compared to basic buffers due to its predominant existence in an ionized form (more soluble). At a certain pH point between pH 1.2 and 9, both the acidic and basic functional groups of RLX may be ionized simultaneously, resulting in its highest solubility. Our study findings also show that RLX solubility increases as pH increases up to 4.5, followed by a gradual decrease in solubility at pH 5.5, 6.8, 7.4, and 8. Furthermore, it is also hypothesized and expected from its p*K*a that RLX would be highly soluble in strongly basic solutions with a pH >11. However, we did not measure its solubility in a strong base as a pH above 8 has limited physiological relevance.

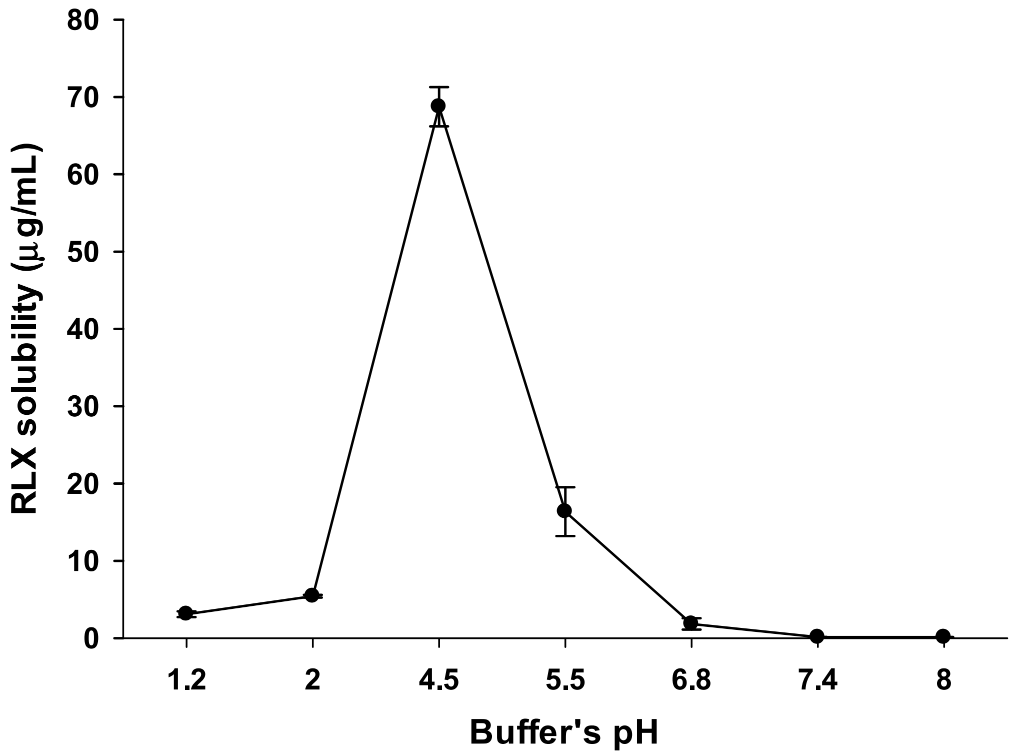

Figure S1. pH-solubility profile of RLX (n = 3).

2. Validation of LC-MS/MS method

The LC-MS/MS method based on the optimized MRM and chromatographic conditions was partially validated in terms of linearity, intraday accuracy and precision, total recovery, extraction efficiency, and matrix effects to confirm the adequacy and suitability of the method for RLX quantification in plasma samples. For method validation, quality control (QC) samples were prepared in rat plasma at four QC levels including the lower limit of quantification (LLOQ, 2 ng/mL), low QC (LQC, 6 ng/mL), mid QC (MQC, 30 ng/mL), and high QC (HQC, 400 ng/mL), followed by the addition of IS solution and extraction as mentioned for the plasma samples and calibration standards. The guidelines of the United States Food and Drug Administration (US-FDA) and the European Medicines Agency (EMA) were followed for method validation as described previously [2-4]. The linearity of the assay was evaluated from the calibration curve constructed by plot[]ting RLX to IS peak area ratios against the respective nominal concentrations of RLX standards. The correlation coefficient (r) value of ≥0.990 was considered acceptable to demonstrate the linearity of the calibration curve [5]. The accuracy and precision of the method were determined by using QC samples at four concentration levels: LLOQ (2 ng/mL), LQC (6 ng/mL), MQC (30 ng/mL), and HQC (400 ng/mL). For intraday accuracy and precision, six replicates of each QC level were quantified within one day. The accuracy, relative error

(%RE) for accuracy, and the precision expressed as the coefficient of variation (%CV or %RSD) were calculated by using the following equations.

$$Accuracy\ (\%) = \frac{Mean\ measured\ concentration}{Nominal\ concentration} \times 100$$

$$RE\ (\%) = \frac{Measured\ concentration\ - Nominal\ concentration}{Nominal\ concentration} \times 100$$

$$Precision\ (CV, \%) = \frac{Standard\ deviation\ of\ measured\ concentations}{Mean\ of\ measured\ concentrations} \times 100$$

The acceptable limits for accuracy and precision were set as %RE and %CV within 15% for LQC, MQC, and HQC levels and within 20% for LLOQ [4]. The total recovery and extraction efficiencies were assessed to ensure that the analyte extraction process is efficient and reproducible, whereas matrix effect was determined to evaluate the impact of plasma components on the ionization process of RLX. For total recovery, extraction efficiency, and matrix effects, the analyte responses (peak areas) were measured using five replicates of LLOQ, LQC, MQC, and HQC. Each QC concentration was prepared in three sets including analyte extracted plasma (analyte added to blank plasma before extraction), analyte spiked into extracted blank plasma (analyte added after extraction of blank plasma), and neat solution of analyte in methanol. Reponses from each of these sets were used to calculate recovery, extraction efficiency, and matrix effects as follows.

$$Total\ recovery\ (\%) = \frac{Peak\ area\ of\ analyte\ extracted\ plasma}{Peak\ area\ of\ \text{analyte solution in methanol}} \times 100$$

$$Extraction\ efficiency\ (\%) = \frac{Peak\ area\ of\ analyte\ extracted\ plasma}{Peak\ area\ of\ \text{analyte into extracted plasma}} \times 100$$

$$Matrix\ effect\ (\%) = \frac{Peak\ area\ of\ \text{analyte into extracted plasma}}{Peak\ area\ of\ \text{analyte solution in methanol}} \times 100$$

The relative matrix effect for each QC sample was determined as the variability (CV, %) in the peak areas of analyte into extracted plasma.

Table S1. Intraday accuracy and precision of the analytical method for the quantification of RLX in rat plasma.

Nominal concentration (ng/mL)	Measured concentration (ng/mL)	Accuracy (%) [RE, %]	Precision (CV, %)
2	2.15 ± 0.07	107.58 [7.58]	3.17
6	6.02 ± 0.06	100.39 [0.39]	0.95
30	28.55 ± 1.29	95.17 [4.83]	4.52
400	418.67 ± 5.05	104.67 [4.67]	1.20

Measured concentrations are presented as the mean ± S.D. (n = 6).

Table S2. Recovery, extraction efficiency and matrix effects for RLX in rat plasma.

RLX concentration (ng/mL)	Total recovery (%)	Extraction efficiency (%)	Absolute matrix effect (%)	Relative matrix effect (CV, %)
2	90.75 ± 2.39	96.35 ± 4.44	94.28 ± 2.98	3.07
6	91.48 ± 1.31	94.76 ± 2.05	98.58 ± 2.56	1.75
30	97.27 ± 4.77	95.00 ± 3.18	102.54 ± 7.10	2.26
400	99.25 ± 3.88	96.34 ± 2.66	103.09 ± 5.20	1.09

Data are presented as the mean ± S.D. (n = 5).

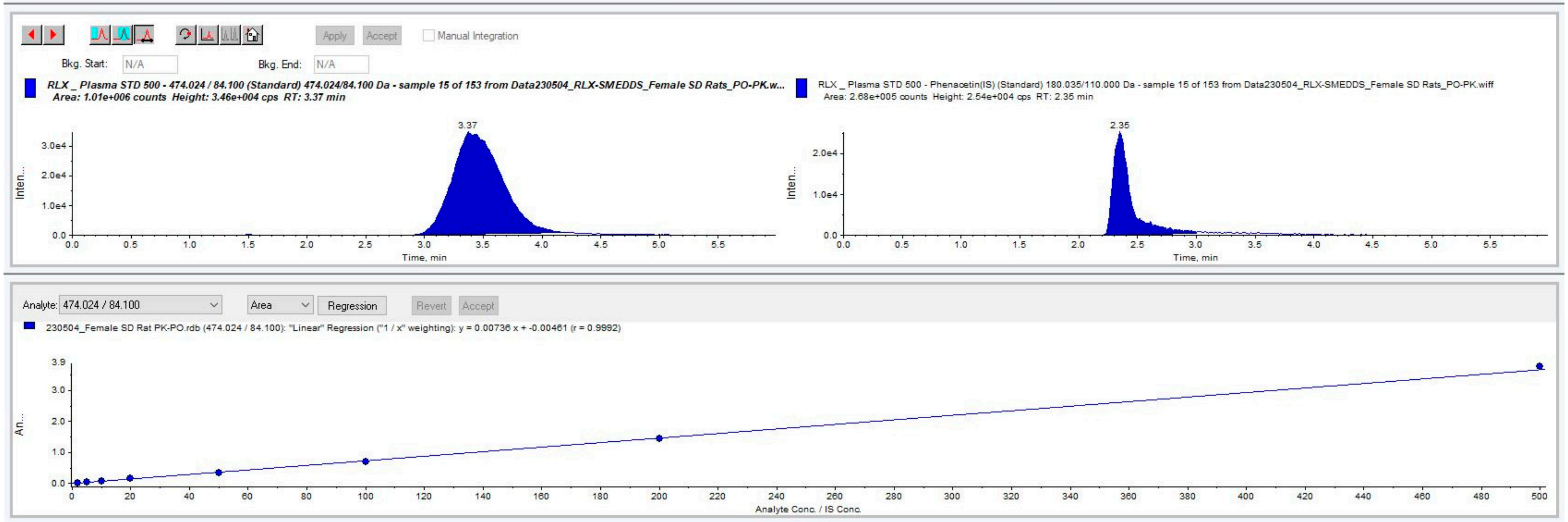

Figure S2. Calibration curve for RLX in the concentration range of 2–500 ng/mL).

References supplementary material

1. Teeter, J.S.; Meyerhoff, R.D. Environmental fate and chemistry of raloxifene hydrochloride. *Environ. Toxicol. Chem.* **2002**, *21*, 729-736.
2. Nguyen, T.-T.-L.; Duong, V.-A.; Vo, D.-K.; Jo, J.; Maeng, H.-J. Development and Validation of a Bioanalytical LC-MS/MS Method for Simultaneous Determination of Sirolimus in Porcine Whole Blood and Lung Tissue and Pharmacokinetic Application with Coronary Stents. *Molecules* **2021**, *26*, 425.
3. ICH. ICH Guideline M10 on Bioanalytical Method Validation ang Study Samples Analysis; European Medicines Agency, International Council for Harmonization: Amsterdam, Netherlands, 2019. Available online: https://www.ema.europa.eu/en/documents/scientific-guideline/ich-guideline-m10-bioanalytical-method-validation-step-5_en.pdf (accessed on 25 June 2023).
4. US-FDA. FDA Guidance for Industry: Bioanalytical Method Validation; Center for Drug Evaluation and Research: Rockville, MD, USA, 2018. Available online: https://www.fda.gov/downloads/drugs/guidances/ucm070107.Pdf (accessed on 25 June 2023).
5. Nguyen, T.-T.-L.; Kim, J.W.; Choi, H.-I.; Maeng, H.-J.; Koo, T.-S. Development of an LC-MS/MS Method for ARV-110, a PROTAC Molecule, and Applications to Pharmacokinetic Studies. *Molecules* **2022**, *27*, 1977.
